# Tuberculin Skin Test Conversion among Individuals with Human Immunodeficiency Virus Infection on Antiretroviral Therapy in a Referral Teaching Hospital, Tehran, Iran

**Published:** 2017

**Authors:** Mahshid Talebi-Taher, Ladan Abbasian, Seiedeh Nina Alavi-Niakou, Seied Ali Javad-Moosavi, Seyedmahdi Pahlavani

**Affiliations:** 1 Antimicrobial Resistance Research Centre, Infectious Diseases Department, Faculty of Medicine, Iran University of Medical Sciences, Tehran, Iran.; 2 Iranian Research Centre of HIV/AIDS, Iranian Institute for Reduction of High-Risk Behaviors, Tehran University of Medical Sciences, Tehran, Iran.; 3 Internal Medicine Department, Faculty of Medicine, Iran University of Medical Sciences, Tehran, Iran.

**Keywords:** Tuberculin skin test, conversion, HIV-positive individuals, Iran

## Abstract

**Background:**

The risk of tuberculosis (TB) is greater for individuals with human immunodeficiency virus (HIV) who are on combined antiretroviral therapy (c-ART) than for the normal population. Therefore, the detection and treatment of latent tuberculosis infections is recommended for all HIV-positive persons with positive tuberculin skin tests (TSTs).

**Materials and Methods:**

This retrospective cohort study included all HIV-positive individuals with CD4 lymphocyte counts greater than 200 cells/μL and negative TST results, who were taking antiretroviral drugs and had been referred to Imam Khomeini Teaching Hospital Consultation Centre for Clients with Risky Behaviors in Tehran, Iran, from 2008 to 2013. TST conversion to positivity is defined as an induration increase of at least 5 mm compared with a previously negative TST result within a 1-year period. Conversion rates are expressed in person-years of observation.

**Results:**

A total of 113 patients were included in our study. At 1 year, 9 of the 113 TST-negative patients taking c-ART became TST-positive (8%; 8 males and 1 female). The TST conversion incidence rate was 10.09/100 person-years. TST conversion was only found to be associated with sex (odds ratio: 8.64; 95% confidence interval: 1.04–7.56, p = 0.032).

**Conclusion:**

Our results suggest that TSTs should be administered to all HIV-positive patients before beginning isoniazid preventive therapy in Iran.

## INTRODUCTION

Tuberculosis (TB) is a leading cause of death in developing countries, especially among HIV-positive people. HIV-infected individuals are at a higher risk for reactivation of latent TB infections or new TB infections than are healthy people; furthermore, the clinical presentation of TB in HIV-infected persons is more complicated (1,2). In a regional study, 7.2% of HIV-infected persons in Iran were co-infected with TB (3). According to a 2010 statement from the Iranian Ministry of Health and Medical Education, a total of 10,485 TB infections were reported in Iran, with 230 (2.2%) of the individuals also being HIV-positive (4). Accordingly, the early diagnosis of latent TB and the prevention of active TB by isoniazid are critical for HIV-infected individuals (5). Isoniazid preventive therapy (IPT) for 6–9 months reduces the risk of TB reactivation among HIV-infected persons by 40–70% (6).

The lifetime risk of TB reactivation is 20% among HIV-infected people, which is defined by a 10-mm or greater induration in a tuberculin skin test (TST). A TST reaction, using purified protein derivative (PPD) in the Mantoux technique, is useful method for latent tuberculosis infection (LTBI) screening (7). However, this test is reported to be less sensitive among HIV-positive patients with low CD4 positive lymphocyte counts (8).

This study aimed to evaluate the TST reaction and its yearly conversion in HIV-infected persons who had CD4 counts ≥200 cells/μL and were TST-negative at the beginning of combined anti-retroviral therapy (c-ART) in a Consultation Centre for Clients with Risky Behaviors in Imam Khomeini Teaching Hospital.

## MATERIALS AND METHODS

This retrospective cohort study included all HIV-infected individuals with CD4 lymphocyte counts greater than 200/μL and negative TST results who were taking antiretroviral drugs and presented to our center between 2008 and 2013. History-taking and physical examination were performed by experienced physicians. Demographic data including age, sex, the possible route of infection, CD4-positive lymphocyte counts, TST results, and chest radiography findings (e.g., fibrous scarring, apical pleural thickening, and calcified nodule) were recorded. We excluded individuals with diabetes mellitus, end-stage renal disease, or chronic liver disease; individuals who were taking immunosuppressive drugs; and patients younger than 13 years of age.

In our clinic, TSTs were performed for all new patients with HIV infection. If a TST result was negative, we repeated the test in 1 year. All results were recorded. We performed the TSTs using the Mantoux technique, with 0.1 mL of PPD (Human Tuberculin 5TU, Razi Vaccine & Serum Research Institute, Iran) administered intradermally. The dermal reaction was interpreted by an expert nurse or physician after 48–72 hours. A positive TST result was defined as an induration of ≥5 mm. A positive TST conversion was defined by a ≥5-mm increase in induration compared with a previously negative TST result in a period of 1 year.

Statistical analysis was performed using SPSS 18 statistical software (SPSS; Chicago, IL). Results are expressed as means ± standard error (SE). The chi-square test and t-test were used for analytical comparison. A *p* value of <0.05 was considered to be significant. Conversion rates are expressed in person-years of observation.

This study was approved by both the Deputy Research of Iran and Tehran Universities of Medical Sciences (NO. 1399).

## RESULTS

From 2008 to 2011, we evaluated 550 individuals with HIV infection. A total of 437 individuals were excluded: 194 patients had CD4-positive lymphocyte counts lower than 200 cells/μL, 52 patients were not receiving c-ART, 29 patients were younger than 13 years of age, 25 patients did not have complete records, 16 patients received c-ART in other centers, 23 patients had active tuberculosis, and 98 patients had positive TSTs at baseline. In all, 113 patients were included in our study.

The mean age of the patients was 37.82 ± 9.11 years. Demographic data are shown in Table 1. At 1 year, 9 of the 113 (8%) TST-negative patients turned TST-positive after taking c-ART. The incidence rate for TST conversion was 10.09/100 person-years. The baseline patient characteristics for TST converters and non-converters are shown in Table 2.

**Table 1.** Demographic data

**Characteristics**	**Patients(n=113)** **N(%)**
**Gender**	
Male	58(51.3)
Female	55(48.7)
**Marital status**	
Single	27(25,2)
Married	69(64.5)
Divorced	7(6.5)
Widow	4(3.7)
Unknown	6(5.3)
**Possible route of transmission**	
Sexual	50(44.24)
Addiction	37(32.74)
Mixed or unknown	26(23.00)

**Table 2.** Univariate analysis of factors associated with TST conversion

Variable	**converters**	**non-converters**	n(%)*	***p* value**	**OR(95%CI)**
**Sex**					
Female	1(1.8)	54(98.2)	55(48.7)	0.032	8.64(1.04–7.56)
Male	8(13.8)	50(86.2)	58(51.3)		
**Chest X-Ray**					
Normal	7(8)	81(92.0)	88(89.8)	0.592	
Abnormal	1(10.0)	9(90.0)	10(10.2)		
**HX. Of IPT**					
Yes	0(0.0)	4(100)	4(3.7)	0.99	
No	8(7.7)	96(92.3)	104(96.3)		
**Hx. of TB treatment**					
Yes	0(0)	1(100)	1(0.9)	0.99	
No	9(8.2)	101(91.8)	110(99.1)		
**Baseline CD4 cell count, cells/μl mean(SD)**	363.11(124.24)	357.55(153.28)	113	0.90	
**CD4 cell count After 6 months of initiation of c-ART, mean(SD)**	464.55(216.16)	388.18(143.01)	113	0.32	
**CD4 cell count After one year of Initiation of c-ART, mean(SD)**	370.57(174.35)	455.91(140.57)	113	0.24	

* **we had some missing data**

In our study population, there was no association between CD4 cell count increment, baseline CD4 cell count, and TST conversion. Of the demographic characteristics, only female sex was found to be associated with TST conversion. Radiologic findings, history of IPT, and history of TB disease/treatment were not associated with TST conversion. CD4 cell count and its increment after 12 months were significantly different between females and males (Table 3).

**Table 3.** Comparing CD4 cell count increase in males and females

	**Male(n=56)**	**female(n=54)**	***p* value**
**CD4 cell count, cells/μl, at baseline, mean(SD)**	328.96(133.91)	388.12(161.99)	0.039
**CD4 cell count, cells/μl, after 6 months of initiation of c-ART, mean(SD)**	363.23(115.21)	425.63(174.45)	0.029
**CD4 cell count After one year of Initiation of c-ART, mean(SD**	396.0.8(143.59)	498.83(124.59)	0.001

All of our patients had a history of Bacille Calmette-Guerin vaccination. No patients developed active TB during the 1-year follow-up period.

## DISCUSSION

In this study over a period of 12 months after c-ART initiation, 9 of 113 (8%) TST-negative patients experienced TST conversion—an incidence rate of 10.09/100 person-years. We found a lower rate of TST conversion than other studies (6,7). These conversions were not related to the reversal of anergy due to c-ART because the CD4 count in our study population was >200 cells/μL at baseline.

Fisk and colleagues demonstrated that TST is a reliable tool for identifying LTBI if CD4 cell counts are >100 cells/μL (9). In our study, neither the baseline CD4 cell count nor the CD4 increase during the 1 year after treatment initiation was found to be associated with TST conversion. Our study found no statistically significant differences between history of IPT or TB treatment and TST conversion rate (*p* = 0.99). However, none of the patients with a positive history of IPT and TB treatment were in the converter group, so this association should be further investigated in a large-scale study. The direct effects of IPT for preventing disease after household contact with a TB-infected individual have been reported previously (10). A meta-analysis found that IPT reduced the risk of active TB by 64% among adults with positive TSTs (11). However, trials evaluating the long-term effects of IPT on PPD conversion rates in HIV infection are needed. We did not have data on other risk factors for LTBI, such as close contact with patients with active TB and a history of incarceration. The lack of this information is a weak point of our study.

Our study found no association between chest radiograph abnormalities and PPD conversion rates (odds ratio: 0.77, 95% confidence interval: 0.08–7.05, p = 0.59), which is in line with a study from Uganda (6). In another study, the majority of participants with positive TST results had a negative chest radiograph; these authors concluded that chest radiography does not demonstrate the benefit of IPT for clinicians (12). To the best of our knowledge, no studies have shown that an abnormal chest x-ray is a risk factor for PPD conversion.

In contrast to a previous study (6), we found that the rate of PPD conversion was significantly higher for males (Table 2). Female sex was independently associated with negative TSTs among HIV-infected adults in Thailand (13). Another study reported that 66–75% of patients with TB in 12 Asian countries were male; therefore, men may account for approximately two-thirds to three-quarters of the burden of TB disease in Asia (14). In China, Gao et al. found that LTBI rates were higher in men than in women (*p* < 0.0001) (15). These reports indicate that men may be more susceptible to TB than women. It remains unclear if male sex is a risk factor for TB. In addition to behavioral factors (e.g., smoking, alcohol, and drug use), biological components also should be considered (16). One study indicated that estradiol acts as an immunity-enhancing mediator in women, with testosterone acting as a mediator inhibiting the immune response (17).

In line with a previous study (18), the women in our study had higher mean CD4 cell counts than men after 1 year of antiretroviral therapy (*p* < 0.001). More frequent side effects were also reported for all classes of antiretrovirals in women (19). Other studies concluded that virologic suppression was achieved more rapidly in women and the response was more durable; therefore, women may experience more benefits for outcomes than men (20, 21).

Oni et al. reported an 8.5% prevalence rate for asymptomatic TB disease among HIV-infected persons. The authors showed that 61% of patients with subclinical TB had a CD4 count of >200 cells/μL; however, a low CD4 count appeared to be predictive of subclinical TB (22). The TST conversion in some of our patients could be due to subclinical active TB. All of our participants were screened for TB symptoms (cough, fever, weight loss, and night sweats). However, we did not collect sputum samples for microscopy and culture, which is a weak point of our study.

Some studies reported that IPT caused a greater reduction in the risk of active TB for TST-positive HIV-infected adults than in TST-negative adults (11,23). In our study, we found a low incidence of TST conversion. Therefore, we recommend that TSTs be performed for all HIV-positive patients before initiating IPT because the increase in CD4 following c-ART may affect TST results. In light of the World Health Organization recommendation on IPT for HIV-positive individuals, clinicians in Iran should consider the local condition and perform TSTs for all HIV-infected patients.
